# Aging and the perception of global structure

**DOI:** 10.1371/journal.pone.0233786

**Published:** 2020-05-29

**Authors:** J. Farley Norman, Alexia J. Higginbotham

**Affiliations:** 1 Department of Psychological Sciences, Ogden College of Science and Engineering, Western Kentucky University, Bowling Green, Kentucky, United States of America; 2 Center for Applied Science in Health and Aging, Western Kentucky University, Bowling Green, Kentucky, United States of America; 3 School of Psychology, University of Southampton, Southampton, United Kingdom; University of Bologna, ITALY

## Abstract

A single experiment required 40 younger and older adults to discriminate global shape as depicted by Glass patterns (concentric and radial organizations). Such patterns have been widely used for decades, because in order to successfully perceive the depicted shape, the visual system has to detect both locally oriented features (dipoles) and their alignments across extended regions of space. In the current study, we manipulated the number of constituent dipoles in the stimulus patterns (40 or 200), the noise-to-signal ratio (zero, 1.0, & 5.0), and the pattern size (6.0 & 25.0 degrees visual angle). The observers’ shape discrimination accuracies (d’ values) decreased markedly as the amount of noise increased, and there were smaller (but significant) effects of both overall pattern size and the number of stimulus dipoles. Interestingly, while there was a significant effect of age, it was relatively small: the overall d’ values for older and younger adults were 2.07 and 2.34, respectively. Older adults therefore retain an effective ability to visually perceive global shape, even for sparsely-defined patterns embedded in noise.

## Introduction

**Fig 1:**
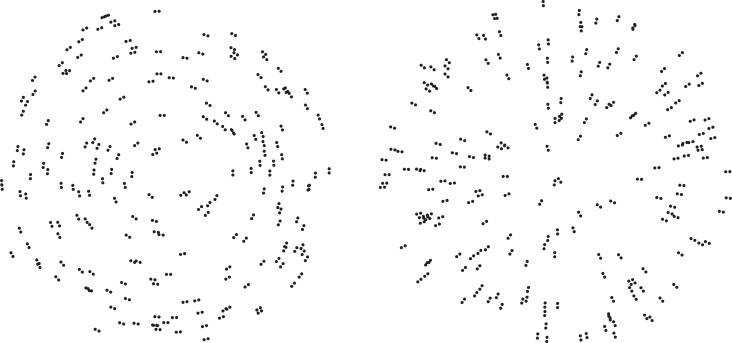
Example stimulus patterns: Glass patterns [1–2] with concentric and radial organizations are shown on the left and right, respectively.

Vision scientists have used Glass patterns [1–2] for decades to investigate the ability of human observers to perceive global shape [3–10]. Examples of radial and concentric Glass patterns are shown in Fig 1. In order to successfully perceive the shapes depicted in Glass patterns, the individual oriented dipoles (pairs of points) must first be detected by neurons within primary visual cortex. Following detection, the local orientations must be integrated across space (e.g., the Boundary Contour System of Grossberg & Mingolla [11–12]) to complete the extended contours that enable shape discrimination.

It is important to investigate aging, because it significantly affects shape perception and other essential visual abilities that support everyday behavior. Any effects of aging upon vision, however, depend greatly on the particular task. For some visual spatial tasks involving the perception of distance, older adults perform well, either better than or the same as younger adults [13–17]. Similarly, older adults can effectively perceive orientation well, especially once age-related differences in contrast sensitivity are taken into account [18–20]. In contrast, while the performance of older adults may be good under certain circumstances [21], adverse effects of age [18,22–33] are often found for tasks involving motion (e.g., motion direction judgment, speed discrimination, perceived shape from motion, biological motion perception, etc.). Tasks that require contour integration also produce age-related deficits [34–35].

McKendrick and colleagues [36–38] have demonstrated that there is a statistically significant adverse effect of increasing age upon the ability to perceive global structures depicted by Glass patterns, but we know little else. Weymouth and McKendrick [38] found, for example, that older adults needed more coherence than younger adults to reliably discriminate between concentric and radial Glass patterns (coherence thresholds were 16.2 and 28.6 percent for younger and older participants, respectively). In the experiments by McKendrick and colleagues, only small diameter Glass patterns were used (5 degrees visual angle for the 2013 studies and 10 degrees visual angle for the 2012 study). If the perception and discrimination of Glass pattern structure depends upon long-range contour completion [11–12] and if there is already a statistically significant effect of age for small diameter patterns (requiring spatial integration covering only relatively short distances), then one might expect that older adults would be even more impaired if they were asked to discriminate global shape over even larger regions of visual space. This is because the visual systems of older adults have particular difficulty with spatial integration [39] and long-range spatial interactions. Consider, for example, the receptive fields of motion-sensitive neurons in cortical area MT [40–41]. The activity of MT neurons can be either suppressed or facilitated by movement within a large spatial area surrounding their classically-defined receptive fields. The modulatory surrounds are very large, up to 50 or 100 times as large as the classical receptive fields. Such center-surround interactions concerning the visual perception of motion can be demonstrated psychophysically in behavioral experiments [26, 42–43]. Such evidence, both neuronal and psychophysical, indicates the existence of long-range spatial interactions within visual mechanisms. A study by Betts, Taylor, Sekuler, and Bennett [44] demonstrated that aging reduces the effectiveness of such long-range spatial interactions. In younger observers [42], increasing the size of a moving high-contrast pattern makes it more difficult to judge motion direction (because the increases in pattern size, past a certain point, stimulate the surround and cause suppression). Betts et al. [44] showed that the effectiveness of this spatial suppression is reduced or eliminated in older adults. An examination of their Fig 2G [44] shows that while the performance of their younger adults was hurt by increases in pattern size, this did not occur for the older adults. It is now clear that changes in the size of visual patterns can have different effects in younger and older adults, because of age-related reductions in the effectiveness of long-range spatial interactions (e.g., suppression) within visual mechanisms.

**Fig 2:**
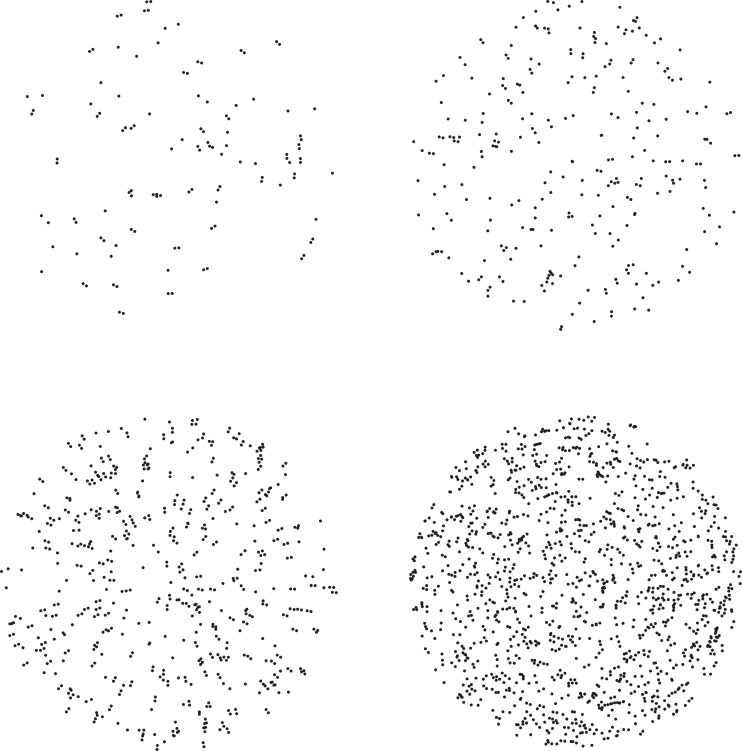
Example Glass patterns with added noise. The top row depicts example stimuli defined by 40 dipoles, while the bottom row depicts example stimuli defined by 200 dipoles. The left column illustrates equal numbers of stimulus dipoles and noise points, while the right column portrays stimulus patterns with 5 times more noise points than signal dipoles.

One important goal of the current experiment was to evaluate younger and older observers’ performance for small (6 degrees, similar to previous stimuli [36–37]) *and* large Glass Patterns (25 degrees). In addition, in the previous studies by McKendrick and colleagues [36–38], coherence thresholds were measured. In the current study, we wanted to determine younger and older observers’ actual perceptual sensitivities to variations in the global shapes depicted by Glass patterns: we therefore used signal detection methodology [45].

## Methods

### Apparatus

The Glass patterns were generated by an Apple dual-processor (1.42 GHz) PowerMacintosh G4 computer and displayed using a 22-inch Mitsubishi Diamond Plus 200 color monitor (1280 x 1024 pixels). The stimulus rendering was accelerated by a Radeon 9000 graphics accelerator (ATI Technologies, Inc.) and hardware antialiasing was employed.

### Experimental stimuli

The experimental stimuli were radial and concentric Glass patterns (white points against a black background) like those used in previous research. The global radial and concentric structure (Fig 1) was defined by either 40 or 200 oriented dipoles (i.e., pairs of points). The individual dipoles on any given trial were randomly placed within a circular area that was 25.0 cm in diameter. The patterns were viewed by the observers either from a distance of 57.3 or 238.5 cm so that the Glass patterns subtended either 25 or 6 degrees visual angle. In one condition, the Glass patterns were presented without noise (i.e., dipoles only), but in other conditions, randomly-placed noise points (noise-to-signal ratios of 1 and 5) were added within the same 25 cm area to obscure the global structure to varying degrees. Examples of Glass patterns containing noise are shown in Fig 2.

### Procedure

To ensure that all observers (both younger and older) clearly understood the task, we presented them with block(s) of 40 trials (20 radial 40-dipole patterns and 20 concentric 40-dipole patterns without noise, presented in a random order) and asked them to indicate whether each pattern depicted a radial or concentric organization. Auditory feedback (a short beep for correct responses) was only presented during practice trials, and was never presented during the experimental trials. The observers were not allowed to begin the actual experiment until after they had performed at least 90 percent correct during a practice block.

Following the practice block, 40 patterns (20 radial and 20 concentric Glass patterns) were judged by each observer for each of the 2 numbers of dipoles (40 and 200), 2 sizes (6 and 25 degrees), and 3 noise-to-signal ratios (0, 1, & 5). Each observer, therefore, made a total of 480 judgments. The trials were blocked by pattern size, thus a block of 240 trials were run for the small patterns (6 deg) and another block of 240 trials were run for the large patterns (25 deg). The order of the size blocks (whether the 6 or 25 degree patterns were judged first) was counterbalanced across observers.

### Observers

There were a total of 40 observers: 20 older adults (mean age was 73.6 years, sd = 5.9, range was 62 to 81 years) and 20 younger adults (mean age was 21.1 years, sd = 2.0, range was 18 to 25 years). The observers’ visual acuity was good (mean acuity for the younger and older adults was -0.05 and 0.01 logMAR, respectively). All observers gave written consent prior to participation in the experiment. The experiment was approved by the Western Kentucky University Institutional Review Board. Our research was carried out in accordance with the Code of Ethics of the World Medical Association (Declaration of Helsinki).

## Results

**Fig 3:**
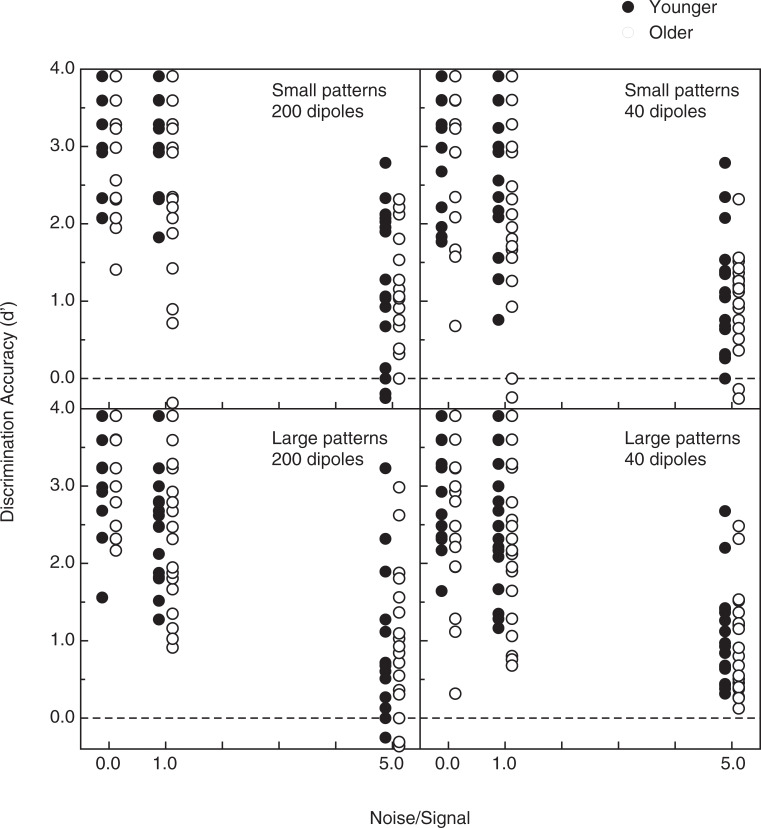
Experimental results. The individual (n = 40) observers’ shape discrimination accuracies (d’) are plotted as functions of 1) the pattern size, 2) the number of signal dipoles, and 3) the amount of noise (relative to signal). The younger observers’ performance is indicated by the filled circles, while the performance of the older observers is indicated by the open circles. The dashed lines indicate chance levels of performance.

The individual observers’ shape discrimination accuracies (d’, see Macmillan & Creelman [45]) are plotted in Fig 3 as functions of the pattern size, the number of signal dipoles, and the amount of noise (relative to signal). It is readily apparent that for any given condition that there was a lot of individual variability in discrimination performance and that the performance distributions for the younger and older observers overlapped extensively. It is interesting to note that even in the most difficult conditions (with a noise-to-signal ratio of 5) that the older observers’ performance was not appreciably lower than that of the younger observers (i.e., there were 8 instances of younger observers’ performing near chance levels at the highest signal-to-noise levels, whereas there were 7 instances of the older observers’ performing near chance levels).

**Fig 4:**
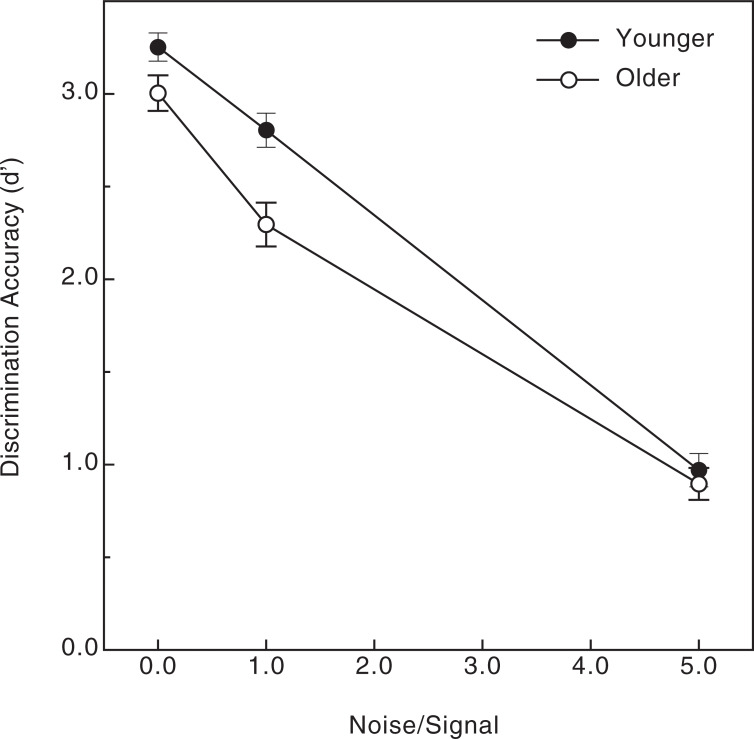
Experimental results. The younger and older observers’ shape discrimination accuracies (d’) are plotted as a function of the amount of noise (relative to signal dipoles). The error bars indicate ± 1 SE.

**Fig 5:**
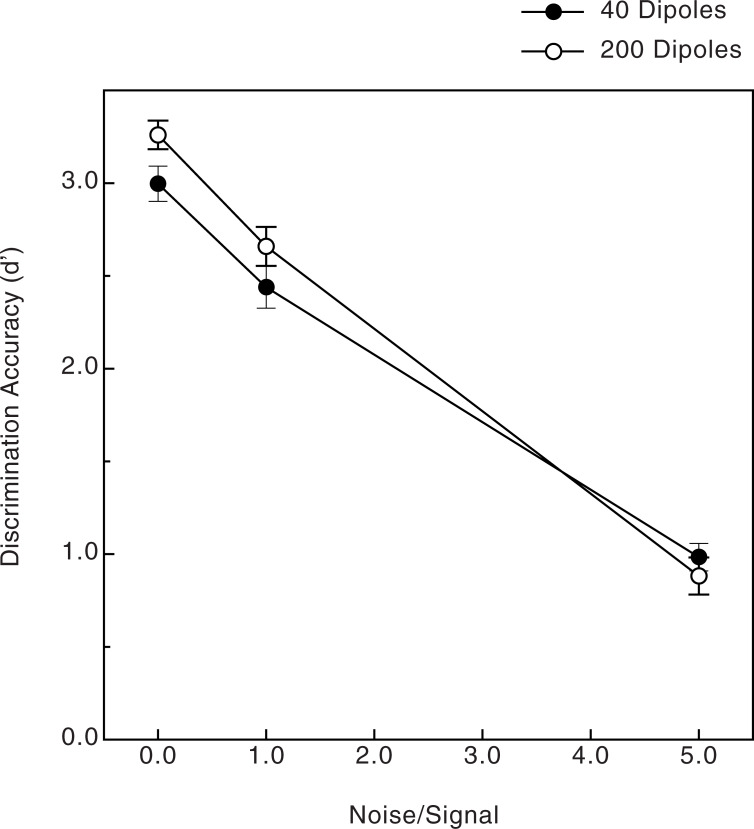
Experimental results. The observers’ shape discrimination accuracies (d’) are plotted as functions of both the number of stimulus dipoles and the amount of noise. The error bars indicate ± 1 SE.

**Fig 6:**
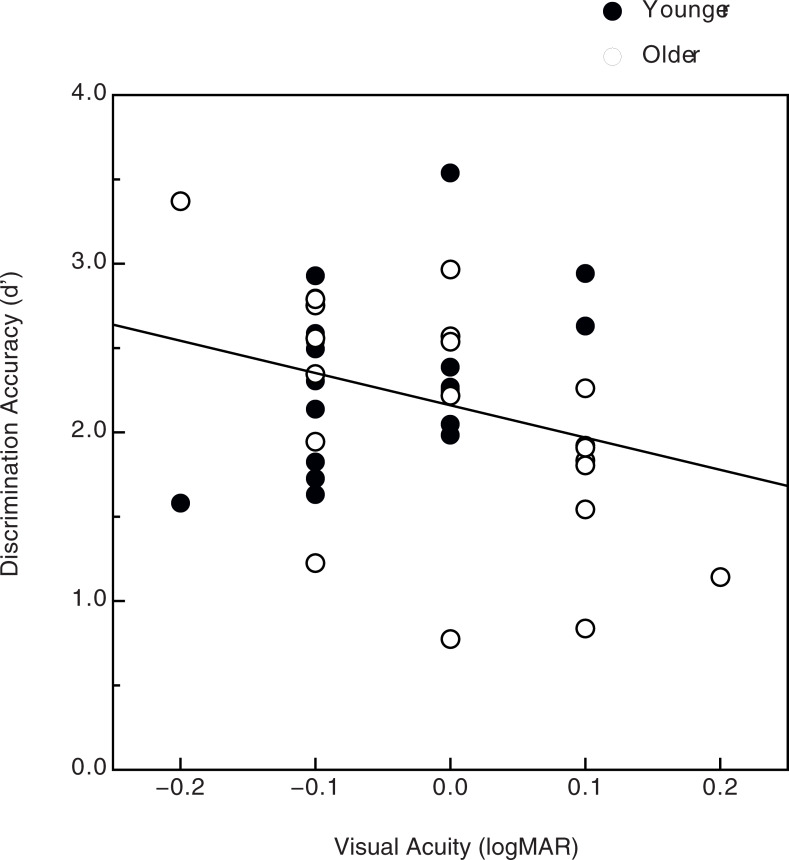
Experimental results. The younger (filled circles) and older (open circles) observers’ average shape discrimination performance is plotted as a function of their individual logMAR (log minimum angle of resolution) visual acuities.

Various aspects of the observers’ results concerning discrimination accuracy are shown in Figs 4–6. The observers’ data (shown in Fig 3) were subjected to a 4-way split-plot analysis of variance (ANOVA, 1 between-subjects factor, age, and 3 within-subjects factors: pattern size, number of dipoles, and amount of noise). First of all, there was a strong effect of noise (Figs 4 & 5, F(2, 76) = 390.1, p < .000001, η^2^_p_ = 0.91): the observers’ perceptual sensitivity to shape was very high (average d’ was 3.0 or greater) when there was no noise and deteriorated substantially as more and more noise points were added to the stimulus patterns. There was a slightly larger effect of noise for the 200-dipole patterns than for the 40-dipole patterns (Fig 5; dipole x noise interaction, F(2, 76) = 4.9, p = .01, η^2^_p_ = 0.11). There was also an adverse effect of age, particularly when moderate amounts of noise were included in the stimulus patterns (i.e., noise x age interaction, see Fig 4: F(2, 76) = 3.6, p = .03, η^2^_p_ = .09). There were small main effects of both the number of dipoles (overall performance for the 200-dipole patterns, d’ = 2.267, was 5.9 percent higher than for the 40-dipole patterns, d’ = 2.140, F(1, 38) = 4.6, p = .039, η^2^_p_ = .11) and pattern size (overall performance for the small patterns, d’ = 2.291, was 8.3 percent higher than for the large patterns, d’ = 2.116, F(1, 38) = 5.8, p = .021, η^2^_p_ = .13). Finally, as Fig 6 indicates, there was a significant relationship between the individual observers’ visual acuities and their shape discrimination performance: as one would logically expect, the lower the observer’s acuity (i.e., higher logMAR value), the lower the average shape discrimination performance (Pearson r = -0.29, p = .03, one-tailed). Even though this relationship between acuity and discrimination performance was significant, the variation in our observers’ acuities accounts for only 8.4 percent (r^2^ = .084) of the variance in their d’ values.

**Fig 7:**
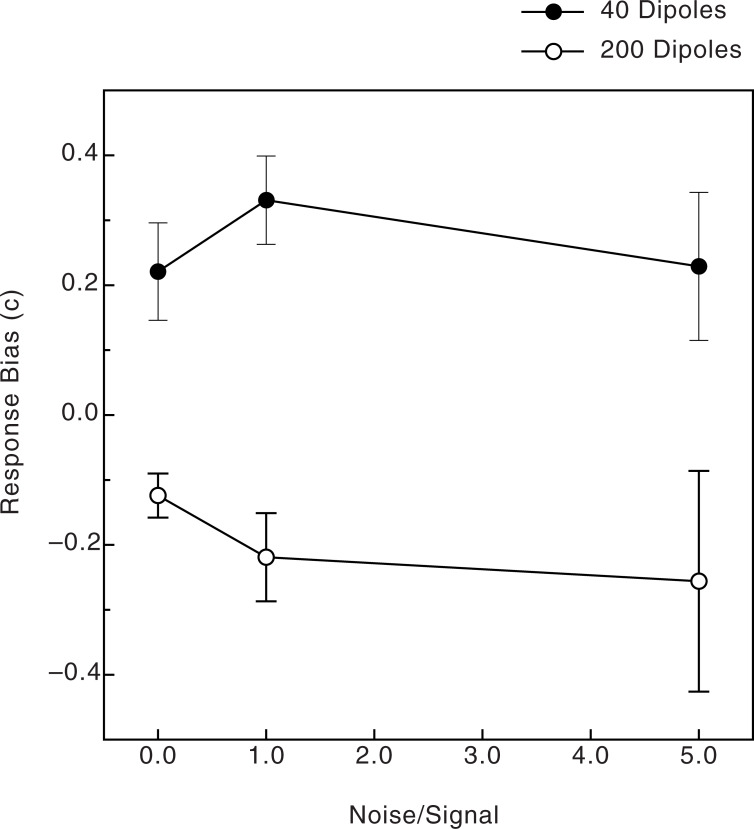
Experimental results. The observers’ response biases (c) are plotted as functions of both the number of stimulus dipoles and the amount of noise. The error bars indicate ± 1 SE.

The observers’ response biases were calculated in terms of c [45] and are shown in Fig 7. While our observers’ response biases were small in absolute terms [45], we nevertheless found a statistically significant effect of the number of dipoles (F(1, 38) = 28.8, p < .00001, η^2^_p_ = .43). As Fig 7 indicates, there was a tendency for the 200-dipole patterns to appear concentric and for the 40-dipole patterns to appear radial. This tendency was largest for the moderate noise condition (i.e., a noise x number of dipoles interaction, F(2, 76) = 3.7, p = .03, η^2^_p_ = .09). There was no difference in obtained response biases between the younger and older observers (no effects of age upon response biases; e.g., no main effect, F(1, 38) = 1.3, p = .27).

## Discussion

In our study, while we found an adverse effect of age, it was relatively modest in size (e.g., the overall d’ values for older and younger adults were 2.07 and 2.34, respectively). The difference in performance between younger and older adults was small for the no-noise condition, and increased in magnitude when moderate amounts of noise were added to the stimulus patterns (see Fig 4). When no noise was included, the older observers performed very well in an absolute sense (d’ of 3.004, which corresponds to approximately 93.3 percent correct [45]): the older observers could effectively discriminate global shape no matter whether the stimulus patterns were relatively small (like those of McKendrick & Battista [36]) or large (25 degrees). When a large amount of noise was introduced into the stimulus patterns (5x more noise than signal) the shape discrimination performances of younger and older adults became quite similar (e.g., the error bars in Fig 4 for younger and older observers overlap in the high-noise condition). When the results are considered as a whole, they clearly demonstrate that the boundary contour system (that enables the perception of global structure in patterns like ours, see Figs 1 & 2) of Grossberg and Mingolla [11] does operate in older adults and functions nearly as well as in younger adults.

In addition to the perception of global structure within Glass patterns, aging also adversely affects performance on related tasks [34–35] that require perceptual completion of extended contours (i.e., contour integration). For example, Roudaia, Bennett, and Sekuler [35] asked younger and older observers to judge the orientation of extended C-shaped contours that were defined by spatially-separated Gabors. The task used by Roudaia et al. was similar to ours in that it required observers to perceive global shape from spatially-separated visual elements. Their younger observers’ performance was facilitated when the Gabors were aligned along the contour path; this facilitation did not occur for the older adults. Roudaia et al. thus concluded (p. 2773) that “contour integration mechanisms in older adults are impaired”. In the current experiment, we also found age-related impairments in global shape perception. It is nevertheless interesting to note, however, that the magnitude of our current age effect seems to be smaller than the one observed previously by Weymouth and McKendrick [38]. Those researchers found that older adults needed 76.5 percent more signal dipoles than younger adults (coherence thresholds were 28.6 and 16.2 percent for their older and younger observers, respectively) to reliably discriminate between concentric and radial Glass patterns that were embedded in noise. Consider the results of our current study (Fig 4) where we also required observers to discriminate between concentric and radial Glass patterns embedded in noise: interpolation shows that the noise-to-signal ratios that would produce threshold performance (d’ = 1.35) for the younger and older observers would be 4.182 and 2.883, respectively. Our older observers, therefore, could tolerate 31.0 percent less noise (4.182–2.883/4.182) than the younger observers when performing at the same level. Even though our study was methodologically quite different from that of Weymouth and McKendrick [38], it appears that our obtained effect of age was perhaps smaller in magnitude than theirs.

It has been demonstrated [46] that neurons in early cortical areas (V1 & V2) are sensitive to, and thus are presumably involved in detecting, the orientations of individual dipoles within Glass patterns. The spatial integration needed to perceive the global forms depicted in Glass patterns occurs in higher visual areas, such as the lateral occipital complex (LOC), as well as V3A and V4 [10, 47–48]. Because the older adults in our study performed relatively well (see Figs 3 and 4), aging apparently does not substantially alter the functionality of higher-order visual areas, such as V4 and the LOC. Additionally, since the effectiveness of such areas (to enable the perception of global shape within Glass patterns) depends critically upon serotonin [9], our results also demonstrate that aging preserves the functionality of the serotonergic system in extrastriate visual cortex.

## Conclusion

Older adults retain an effective ability to visually perceive global shape, even for sparsely-defined patterns embedded in noise. In the current study, we found only a small quantitative effect of age upon sensitivity to perceived shape (i.e., d’ values), depite the fact that our older observers were more than 52 years older (on average) than our younger observers.

## Supporting information

S1 Dataset Younger and older observers’ discrimination accuracies (d’ values). (XLSX)

S2 Dataset Younger and older observers’ response biases (c values). (XLSX)

S1 Table ANOVA summary table for d’ values. (XLSX)

S2 Table ANOVA summary table for c values. (XLSX)
